# Impact of sacubitril/valsartan on cardiac and systemic hypoxia in chronic heart failure

**DOI:** 10.1016/j.isci.2023.108520

**Published:** 2023-11-23

**Authors:** Hélène Nougué, François Picard, Alain Cohen-Solal, Damien Logeart, Jean-Marie Launay, Nicolas Vodovar

**Affiliations:** 1 Université de Paris and Inserm UMR-S 942, Paris, France; 2 Department of Anaesthesiology and Intensive Care, Saint Louis – Lariboisière – Fernand Vidal University Hospital, Paris, France; 3 Service d’insuffisance cardiaque, Hôpital Cardiologique du Haut-Lévêque, Pessac, France; 4 Department of Cardiology, Lariboisière Hospital, Paris, France

**Keywords:** Health sciences, Medicine, Pharmacology

## Abstract

In heart failure patients with reduced ejection fraction, Sacubitril/valsartan (S/V) increased proBNP T71 glycosylation, which is regulated negatively by hypoxia via miR-30a *in vitro*. Using a cohort of 73 HFrEF patients who were transitioned from standard HF medication to S/V, we found that the increase in proBNP T71 glycosylation after S/V was associated with a decrease in cardiac hypoxia. We further found that plasma levels of K709-acteylated HIF1α, HIF-regulated and HIF-independent biomarkers also evolved consistently with a decrease in hypoxia. We further confirmed that biomarker changes were related to hypoxia, in a rat model subjected to isobaric hypoxia. We measured them in rats subjected to isobaric hypoxia. Overall, these data strongly suggest that optimally treated HFrEF patients exhibited subclinical hypoxia that is improved by S/V. The data also posit proBNP T71 glycosylation as a biomarker of cardiac hypoxia.

## Highlights

• Heart failure patients exhibit systemic sub-clinical hypoxia
• Sacubitril/valsartan improves hypoxia systemically
• ProBNP T71 glycosylation appears as a biomarker of cardiac hypoxia

## Graphical abstract

**Figure:**
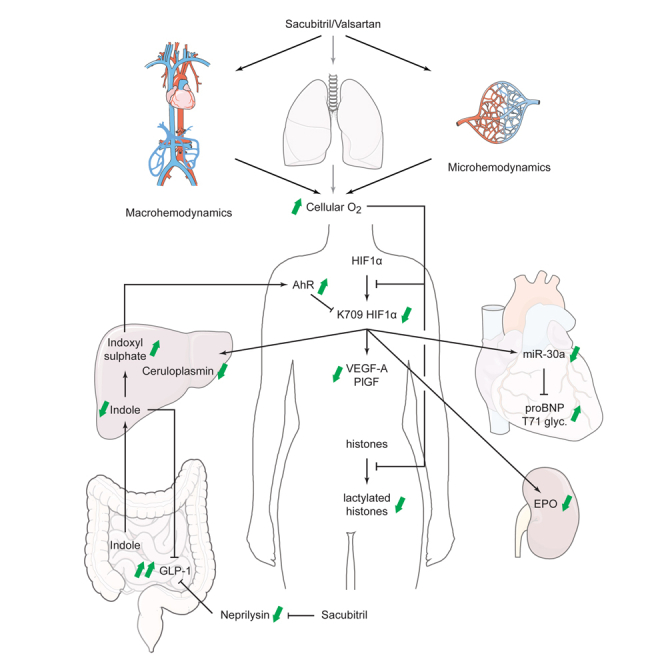

## Introduction

Sacubitril/valsartan (S/V) is the first-in-class combination of angiotensin receptor blocker and neprilysin inhibitor (ARNi) that has markedly improved HF natural history and outcome in patients with heart failure with reduced ejection fraction (HFrEF).^1^ Despite this impressive improvement in outcome, the mode of action of S/V is not fully understood. We previously showed that S/V provoked an increase in proBNP glycosylation at threonine 71 (T71)^2^ between before and D30 after the initiation of S/V. ProBNP T71 glycosylation is initiated by the N-acetylglucosaminyltransferase 1/2 (GalNac-T1/2)^3^ and is restricted to ventricular cardiomyocytes in the heart *in vivo.*^4^ It has recently been shown that GalNac-T1/2 expression is negatively regulated by miRNAs of the miR-30 family,^3^ of which miR-30a was shown to be induced by hypoxia in cardiomyocytes *in vitro*.^5^

Hypoxia is defined as a mismatch between oxygen requirement and delivery to cells. Hypoxia could be attributed to inadequate cardiac output (circulatory hypoxia), inadequate oxygen transport (hypoxemia: decreased oxygen saturation or transport capacity), or inadequate capillary perfusion (microhemodynamic hypoxia). Furthermore, central sleep apnea provokes transient decrease in blood saturation and triggers the expression of hypoxia-regulated factors,^6^ and S/V was shown to be beneficial in this context.^7^^,^^8^ Cells modulate their transcriptional program to respond to hypoxia. The transcriptional response to hypoxia is pleiotropic and modulates the expression of genes involved in metabolism, extracellular matrix remodeling, and cell signaling amongst other processes.^9^ In humans, hypoxia is associated with an increase in renal erythropoietin (EPO) production to regulate erythropoiesis, liver ceruloplasmin production to regulate copper and iron metabolism,^10^ and vascular endothelial growth factor (VEGF) and placental growth factor (PlGF) that are involved in angiogenesis. The response to hypoxia is predominantly transcriptional and is driven by two hypoxia-inducible transcriptional factors (HIF) with overlapping and complementary functions.^9^ HIF transcriptional factors are heterodimers composed of an alpha subunit (HIF1α or HIF2α) that acts as oxygen sensors and a common β-subunit (ARNT: Aryl hydrocarbon Receptor Nuclear Translocator). All subunits are constitutively expressed. HIF1α and HIF2α are hydroxylated and rapidly targeted to the proteasome under normoxic conditions.^11^^,^^12^ Under hypoxic conditions, HIF1α and HIF2α are less hydroxylated, hence less degraded, bind to ARNT and regulate the transcription of their target genes. In addition, the acetylation of HIF1α Lysine 709 (K709ac) increases the expression of HIF1α targets,^13^ and cellular hypoxia increases intracellular lactate production which leads to histone lactylation,^14^ which also contributes to changes in gene expression.

ARNT can also bind to the Aryl hydrocarbon Receptor (AhR), which is mainly involved in the metabolism of xenobiotic compounds.^15^ The sharing of ARNT by AhR and HIFα subunit leads to a competition between the two pathways. Amongst potent AhR activators are indoxyl sulphate, *para*-cresol (*p*-cresol), and *para*-cresyl sulphate (*p*-cresyl-sulphate).^16^ Indole and *p*-cresol are produced by the gut microbiota from tryptophan and tyrosine, respectively. They are eliminated by the kidney after sulphoconjugation as indoxyl sulphate and *p*-cresyl-sulphate; the sulphoconjugation of indole requires a preceding hydroxylation by cytochrome CYP2E1.^17^^,^^18^ In chronic kidney disease, indoxyl sulphate, *p*-cresol and *p*-cresyl-sulphate tend to accumulate in bodily fluids resulting in an exacerbated AhR response. The enhanced AhR response outcompete that of hypoxia and AhR activation by indoxyl sulphate reduced the production of EPO.^19^ Therefore, the response to hypoxia is no longer adapted to the level of hypoxia, contributing to the worsening of kidney disease.

Given the longitudinal nature of the cohort used^2^ and the rapid changes observed in proBNP glycosylation at T71 in this cohort, it offered an opportunity to evaluate the possible role of hypoxia as a mechanism underlying the variation of proBNP T71 glycosylation observed in patients treated with S/V. The results obtained prompted us to evaluate whether the improvement in oxygen bioavailability suggested in the heart also applied to other organs.

## Results

### proBNP glycosylation at T71 is associated with miR-30a *in vivo*

**Figure 1:**
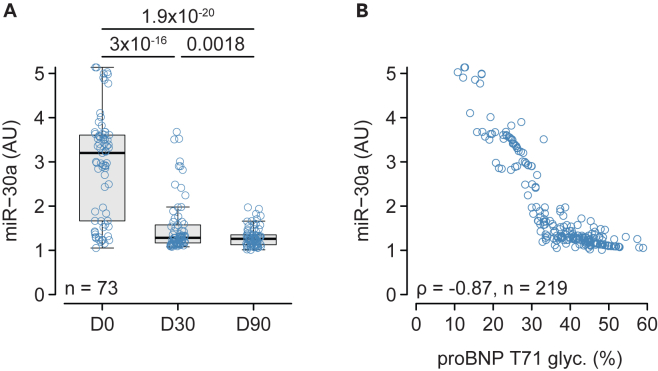
proBNP glycosylation at T71 and miR-30a *in vivo* (A) Plasma levels of miR-30a at baseline (D0), and D30 and D90 after the introduction of S/V. (B) Relationship between plasma miR-30a plasma levels and proBNP T71 glycosylation. Variables in A were analyzed using repeated-measure ANOVA on log-transformed data followed by the paired Student’s *t* test corrected for multiple comparisons (holm). Correlations between variables were estimated with Spearman rank correlation and expressed by the correlation coefficient (ρ). The number of subjects is indicated in each panel.

While proBNP T71 glycosylation increased at D30 and D90 after the introduction of S/V^2^, plasma levels of miR-30a decreased (Figure 1A). This decrease in miR-30a plasma levels was strongly inversely correlated with proBNP T71 glycosylation (Figure 1B). T71 proBNP glycosylation was already increased at D7 (Figure S1A), and there was a trend toward a decrease in miR-30a (p = 0.056, Figure S1B). At D14, T71 was markedly increased and miR-30a was markedly decreased when compared to baseline (Figures S1C and S1D), and the strong negative correlation between proBNP T71 glycosylation and miR-30a was also present at D7 and D14 (Figure S1E).

### HIF-dependent biomarkers of hypoxia

**Figure 2:**
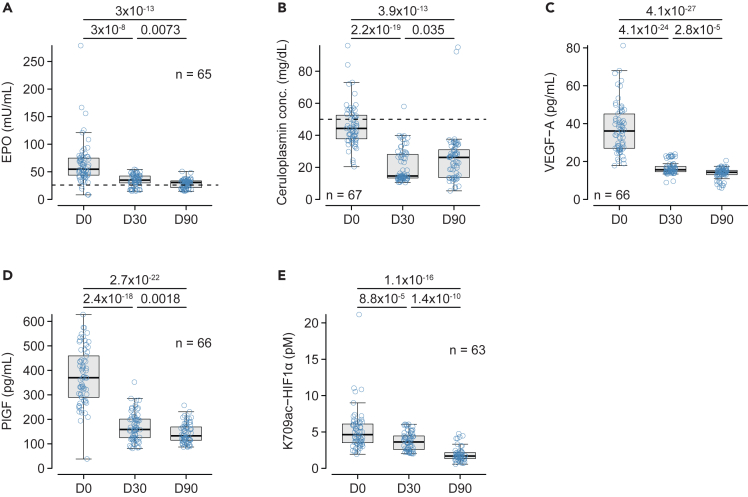
Evolution of HIF1α-dependent biomarkers Plasma concentrations of erythropoietin (EPO, A), ceruloplasmin (B), vascular endothelial growth factor A (VEGF-A, C), placental growth factor (PlGF, D), and HIF1α acetylated on lysine 709 (HIF1a K709ac, E) at baseline (D0), and D30 and D90 after the introduction of S/V. Variables were analyzed using repeated-measure ANOVA on log-transformed data followed by the paired Student’s *t* test corrected for multiple comparisons (holm). The number of subjects is indicated in each panel. The dotted line in A and B indicate the upper limit of the normal range.

The increase in proBNP T71 glycosylation and the decrease in miR-30a suggested a decrease in cardiac hypoxia. To evaluate a possible decrease in hypoxia in other tissue and organs, we measured organ-specific (EPO and ceruloplasmin) and systemic (VEGF-A, PlGF, and K709ac HIF1α) biomarkers that are upregulated under hypoxic conditions. At baseline, 94% of patients had EPO >26 mIU/mL (Figure 2A), and 30% had ceruloplasmin concentration >50 mg/dL (Figure 2B). Both EPO and ceruloplasmin concentrations decreased after the introduction of S/V at D30 and D90, although 70% of patients had EPO levels >26 mIU/mL at D90. The ubiquitous biomarkers of hypoxia – i.e., VEGF-A (Figure 2C), PlGF (Figure 2D), and K709ac HIF1α (Figure 2E), markedly decreased at D30 and D90. Of note, EPO, ceruloplasmin, and PlGF were already decreased at D7, while VEGF-A only decreased at D14. K709ac HIF1α did not decrease at D7 and D14 (Figure S2). Although the biomarkers tested can be transcriptionally regulated by HIF1α, there were only moderate correlations between EPO, ceruloplasmin, VEGF-A, and PlGF concentrations and K709ac HIF1α (Figure S3). The decrease in these hypoxia-related biomarkers was not associated with any change in either hemoglobin, hematocrit, or red blood cell count (Figure S4), indicating that the decrease in the various hypoxia-related biomarkers tested did not result from an increase in oxygen transport capacity. Finally, none of the patients exhibited oxygen saturation below 98% at any time point neither did they exhibit Cheyne-Stokes respirations as a symptom of central apnea.

### HIF-independent biomarkers of hypoxia

**Figure 3:**
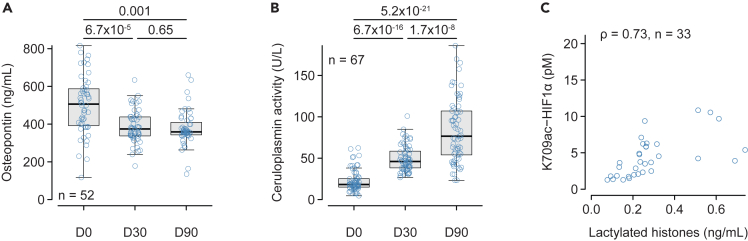
Early evolution of HIF1α-independent biomarkers (A) Plasma levels of osteopontin at baseline (D0), and D30 and D90 after initiation of S/V. (B) Ceruloplasmin ferroxidase activity measured in the plasma at baseline (D0), and D30 and D90 after the introduction of S/V. (C) Relationship between HIF1α acetylated on lysine 709 (HIF1a K709ac) and lactylated histones in a subset of patients. Variables in A and B were analyzed using repeated-measure ANOVA on log-transformed data followed by the paired Student’s *t* test corrected for multiple comparisons (holm). Correlations between variables were estimated with Spearman rank correlation and expressed by the correlation coefficient (ρ). The number of subjects is indicated in each panel.

To further ascertain a decrease in hypoxia rather than an inadequate decrease in the response to hypoxia, we measured markers that are positively regulated by hypoxia in a HIF-independent fashion.^20^ We found that plasma levels of osteopontin decreased (Figure 3A) while the oxygen-dependent ferroxidase activity of ceruloplasmin (EC 1.16.3.1) markedly increased at D30 and D90 (Figure 3B). Finally, we measured plasma levels of lactylated histone, which is a marker of intracellular lactate accumulation, in a limited subset of patients and found it was strongly positively correlated to plasma levels of K709ac HIF1α (Figure 3C).

**Figure 4:**
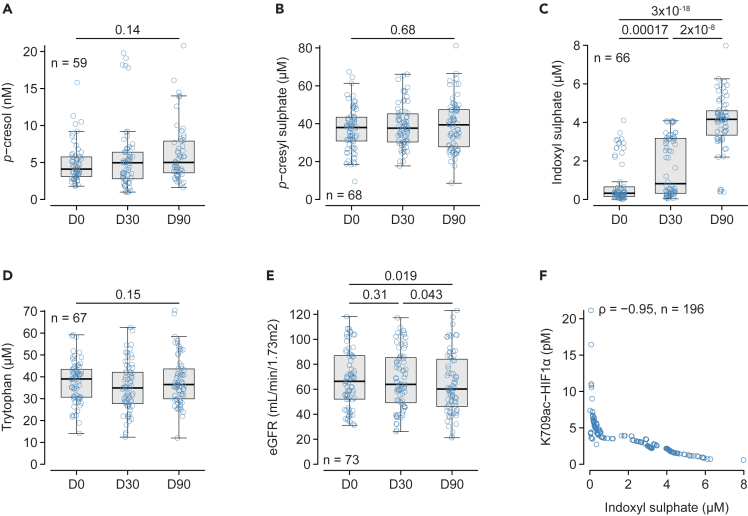
Evolution of para-cresol and indoxyl sulphate Plasma levels of para-cresol (*p*-cresol, A), and its sulphoconjugate para-cresyl sulphate (B) at baseline (D0), and D30 and D90 after the introduction of S/V. Plasma levels of indoxyl sulphate (C) and its precursor tryptophan (D) at baseline (D0), and D30 and D90 after the introduction of S/V. (E) Estimated glomerular filtration rate (eGFR) calculated according to the 2021 revision of the CKD-EPI formula at baseline (D0), and D30 and D90 after the introduction of S/V. (F) Relationship between HIF1α acetylated on lysine 709 (HIF1a K709ac) and indoxyl sulphate. Variables in A-E were analyzed using repeated-measure ANOVA on log-transformed data followed by the paired Student’s *t* test corrected for multiple comparisons (holm). Correlations between variables were estimated with Spearman rank correlation and expressed by the correlation coefficient (ρ). The number of subjects is indicated in each panel.

Another way to evaluate a genuine decrease in hypoxia is to compare the production of *p*-cresyl sulphate and indoxyl sulphate, the latter requiring molecular oxygen to be produced in hepatocytes (Figure S5). The plasma levels of *p*-cresol (Figure 4A) and its sulphoconjugate *p*-cresyl sulphate (Figure 4B) remained unchanged throughout the study. In marked contrast, the plasma levels of indoxyl sulphate increased by 2.9 [0.6–10.8] folds between baseline and D30, and by 3.0 [1.1–12.3] folds between D30 and D90 (Figure 4C), while its precursor, tryptophan, remained unchanged throughout the study (Figure 4D). The increase in indoxyl sulphate was not associated with the worsening of kidney function as eGFR remained unchanged between baseline and D30 and marginally declined between D30 and D90 (Figure 4E). Finally, there was a strong negative correlation between the plasma levels of indoxyl sulphate and K709 HIF1α (Figure 4F).

### Specificity of biomarker changes

To test the specificity of S/V over the up-titration of Valsartan, we first estimated a Valsartan dose-equivalence for all patients treated with ARB and ACEi at baseline (10 mg Ramipril daily = 320 mg Valsartan daily), and for patients treated with S/V at D30 (97 mg/103 mg S/V bid = 320 mg Valsartan daily).^21^ Patients were split into two groups based on whether their Valsartan dose-equivalence was up-titrated between baseline and D30 or not. The variations of all the biomarkers tested were similar between the two groups, suggesting that most of the effects observed are specific to S/V rather than the up-titration of Valsartan alone (Table 1).

**Table 1:** Biomarker relative variations (%) in patients whose Valsartan dose equivalence was not up-titrated or up-titrated between baseline and D30

Variable	Valsartan not up-titrated (n = 44)	Valsartan up-titrated (n = 25)	P
proBNP T71 glycosylation (%)	39 [21.7; 76] (n = 44)	65.8 [29.9; 85.4] (n = 25)	0.29
miR-30a (AU)	−42 [−63.4; −15.6] (n = 44)	−51.6 [−64.7; −20.1] (n = 25)	0.54
Ceruloplasmin conc. (mg/dL)	−64.1 [−70.1; −32.9] (n = 41)	−63.7 [−70.4; −36.5] (n = 23)	0.96
Ceruloplasmin act. (U/L)	168.3 [73.9; 293] (n = 41)	185.5 [64.1; 300.2] (n = 23)	0.97
K709ac HIF1α (pM)	−14.6 [−42.2; 16.3] (n = 40)	−32.7 [−45.6; 19.1] (n = 20)	0.51
EPO (mIU/mL)	−42.9 [−63.6; −20.7] (n = 41)	−34.6 [−55.6; −23] (n = 21)	0.86
VEGF-A (pg/mL)	−51 [−64.4; −43.9] (n = 41)	−48.6 [−67.1; −35.9] (n = 22)	0.99
PlGF (pg/mL)	−59.9 [−71.2; −42.8] (n = 41)	−52 [−61.3; −36.7] (n = 22)	0.09
Indoxyl sulphate (μM)	43.6 [−52; 712.8] (n = 41)	240.7 [−8.8; 1191.2] (n = 22)	0.19
Osteopontin (ng/mL)	−24 [−39.3; −7.4] (n = 28)	−25.1 [−41.4; −1.5] (n = 22)	0.81

Data are represented as median and interquartile range. The number of patients for each comparison is indicated between brackets. K709ac HIF1α: HIF1α acetylated on lysine 709, EPO: erythropoietin, VEGF-A: vascular endothelial growth factor A, PlGF: placental growth factor.

### Biomarker validation in a hypoxic rat model

**Figure 5:**
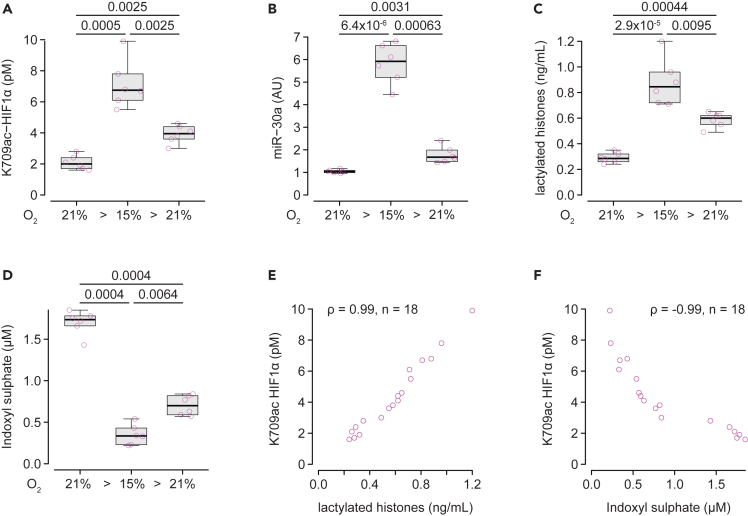
Evolution of hypoxia biomarkers in rats subjected to isobaric hypoxia Plasma levels of HIF1α acetylated on lysine 709 (HIF1a K709ac, (A), miR-30a (B), lactylated histones (C) in rats under normoxic conditions (21% O_2_), after one week in isobaric hypoxia (15% O_2_), and one week after returning to normoxia (21% O_2_). (E) Relationship between HIF1a K709ac and lactylated histones. (D) Plasma level of indoxyl sulphate in rats under normoxic conditions (21% O2), after one week in isobaric hypoxia (15% O2), and one week after returning to normoxia (21% O2). (F) Relationship between HIF1a K709ac and indoxyl sulphate. Variables in A-C and E were analyzed using repeated-measure ANOVA on log-transformed data followed by the paired Student’s *t* test corrected for multiple comparisons (holm). Correlations between variables were estimated with Spearman rank correlation and expressed by the correlation coefficient (ρ). There were 6 rats in the experiment that were followed longitudinally throughout the study.

To fully validate the relationship between the biomarkers tested and hypoxia, we subjected wild-type male rats to isobaric hypoxia (15% oxygen), and measured those biomarkers before, after a week under isobaric hypoxic atmosphere, and after a week back under normal atmosphere. As expected, K709ac HIF1α increased with hypoxia (Figure 5A), which was accompanied by an increase in miR-30a (Figure 5B), EPO (Figure S6A), ceruloplasmin (Figure S6B), and VEGF-A (Figure S6C) plasma concentrations. HIF1α-independent markers lactylated histone (Figure 5C) and osteopontin (Figure S6D) also increased, while ceruloplasmin activity decreased (Figure S6E). Indoxyl sulphate decreased upon hypoxia (Figure 5D), unlike its precursor tryptophan (Figure S6F) and p-cresol (Figure S6G) and p-cresyl sulphate (Figure S6H) which remained unchanged throughout. After placing the rats back in a normal atmosphere for a week, all the markers trended toward baseline levels, although not reaching them. As observed in the cohort, there were strong positive correlations between K709ac HIF1α and lactylated histones (Figure 5E) or indoxyl sulphate (Figure 5F) plasma concentrations.

## Discussion

In this study, we show that HFrEF patients under optimal medication exhibit a moderate subclinical cellular hypoxia in numerous tissues and organs that improved after the introduction of S/V. Importantly, the improvement in hypoxia preceded clinical improvement as measured by the NYHA classification. Furthermore, our data support proBNP T71 glycosylation as a biomarker of cardiac hypoxia.

We found that most of the HFrEF patients included in this study had subclinical hypoxia affecting multiple tissues or organs, even under optimal treatment with HF therapies. The introduction of S/V led to consistent improvements in all biomarkers of hypoxia by day 30, which appears to be specific of the combination of sacubitril and valsartan. However, S/V also markedly increased indoxyl sulphate plasma levels, which could inappropriately blunt the response to hypoxia via AhR activation without any change in oxygenation. However, several lines of evidence suggest that this scenario is unlikely: (1) the increase in indoxyl sulphate likely reflects an increase in oxygen-dependent hydroxylation of indole^17^^,^^18^ as indoxyl sulphate also increased after placing rats back in normoxic conditions after a week under hypoxic conditions; (2) indoxyl sulphate unlikely accumulated due to reduced renal clearance as p-cresyl sulphate, which is cleared by the kidney ∼3 times less effectively by the kidney than indoxyl sulphate,^22^ remained unchanged throughout the study despite a marginal decline in kidney function at D90 only; (3) several HIF-independent yet oxygen-dependent processes (histone lactylation, ceruloplasmin oxidase activity) improved both in humans and rats; and (4) the correlation between lactylated histone and K709ac HIF1α indicate that the decrease in the latter likely results from increased cellular oxygenation. In light of these results, the strong negative correlation between indoxyl sulphate and K709ac HIF1α likely reflects their inverse dependency on oxygen rather than a competition between the two processes, and may also reflect the contribution of AhR in the proper down-regulation of the response to hypoxia when oxygenation has improved.^23^ Also, the indoxyl sulphate/*p*-cresyl sulphate ratio appears as a biomarker of intracellular oxygen availability in hepatocytes when their clearance is not reduced. Of note, some hypoxia-related biomarkers improved at day 7 and day 14 after the introduction of S/V - i.e., before the improvement of symptoms, suggesting that the improvement of tissue perfusion is rapid. However, there was only a trend toward a decrease in plasma mir-30a levels while proBNP T71 glycosylation already decreased. This might reflect the fact that proBNP is secreted while miRNAs are released via exosomes, therefore not necessarily directly reflecting the short-term dynamic of miRNA change inside the cell. The fact that all the biomarkers tested continued to improve at day 90 only reflects the slow shutdown of the response to hypoxia, as observed in rats. Taken together, these results strongly suggest that changes in biomarkers resulted from a true improvement in hypoxia and that S/V rapidly improves tissue perfusion.

Several non-exclusive mechanisms could account for the improvement in oxygen availability observed. Results from the PARADIGM-HF,^1^ PROVE-HF^24^ and EVALUATE^25^ trials suggested that cardiac function and central hemodynamic improved, although EVALUATE did not find any impact of S/V on aortic stiffness.^25^ Alternatively, S/V may improve oxygen availability independently from any hemodynamic effect as it has been observed for the attenuation of renal injury in a rat model of obesity,^26^ or by reducing central apnea.^7^^,^^8^ Finally, it is unlikely that the improvement of hypoxia results from an increase in oxygen transport capacity as neither hemoglobin, hematocrit nor red blood cell count changed throughout the study. Furthermore, patients exhibited normal oxygen saturation and did not show evidence of central apnea, although we did not evaluate apnea during sleep. Therefore, the mechanism whereby S/V reduced tissue hypoxia remains to be characterized but is likely to involve macro- and micro-hemodynamic parameters, as well as possible hemodynamic-unrelated effects. This improvement of hypoxia could also explain changes observed after the introduction of S/V in other biomarkers such as troponin and GLP-1.^2^^,^^27^ In particular, long-term exposure of intestinal enteroendocrine L cells to indole limits the secretion of GLP-1^28^ and the anticipated decrease in indole via its increase in sulphoconjugation after S/V initiation is expected to increase plasma GLP-1 in synergy with its direct protection from NEP degradation by sacubitril.^2^ As observed for ANP,^29^ the effect of S/V on GLP-1 appears to be direct and indirect, and could participate in the metabolic benefit of S/V in HFrEF.^2^^,^^30^ The impact of S/V on hypoxia also echoes the benefit observed in a rat model of pulmonary arterial hypertension,^31^ altogether being encouraging results for the undergoing trials testing S/V in this disease. Taken together, these data strongly suggest that the improvement of tissue perfusion participates in the mode of action of S/V.

Finally, we confirmed *in vivo* the negative relationship between miR-30a and proBNP T71 glycosylation, strongly suggesting that proBNP T71 glycosylation is a biomarker of ventricular cardiomyocyte hypoxia.^4^ Importantly, patients with acute dyspnea of cardiac (ADHF) and non-cardiac (non-ADHF) origins had similar levels of proBNP T71 glycosylation, albeit radically different natriuretic peptide levels.^32^ In non-ADHF patients, lower proBNP T71 glycosylation levels were achieved at lower natriuretic peptide plasma levels than in ADHF patients,^32^ suggesting that ADHF and non-ADHF patients suffer from similar levels of cardiac hypoxia, and that in that instance, natriuretic peptide levels do not reflect the level of hypoxia the heart is subjected to. Taken together, these data strongly suggest that proBNP glycosylation at T71 is a biomarker of cardiac hypoxia in HFrEF and acute conditions.

In conclusion, HFrEF exhibit subclinical tissue/organ hypoxia that is improved by S/V, and which may account for S/V clinical benefit. The cause of this improvement in tissue/organ hypoxia is unknown and could rely on hemodynamic- and non-hemodynamic-related mechanisms that are yet to be identified. More generally, subclinical hypoxia has long been overlooked and could be clinically relevant in the management of chronically and acutely ill patients, although further studies are warranted.

### Limitations of the study

Our study has some limitations. First, the study population is rather small and not randomized which may limit the statistical performance. However, the changes observed in the various parameters tested are consistent across the population. Second, S/V up-titration was left at the discretion of the cardiologist according to the tolerance and patient clinical status, and therefore it is not possible to precisely assess the relationship between dose and time. Third, we did not measure any direct parameters of tissue oxygenation nor did we study respiratory patterns and sleep apnea. However, the methods available can only measure tissue oxygen in the skin non-invasively and skeletal muscle oxygenation invasively. Finally, the open design and the lack of a control arm may have affected the assessment of the clinical response even though the study is longitudinal and each patient is under his/her control.

## STAR★Methods

### Key resources table

**Table:** 

REAGENT or RESOURCE	SOURCE	IDENTIFIER
**Antibodies**		

Rat EPO	Santa Cruz	sc-5290; RRID: AB_627551
Rat ceruloplasmin	Santa Cruz	sc-365205; RRID:AB_10709009

**Biological samples**		

Human EDTA plasma samples	NCT01374880	NCT01374880

**Chemicals, peptides, and recombinant proteins**		

*p*-phenylenediamine	SIGMA	695106; CAS: 106-50-3
Lys-C	Custom Biotech	501839066
Tris(2-carboxyethyl)phosphine hydrochloride (TCEP)	SIGMA	C4706; CAS: 51805-45-9

**Critical commercial assays**		

Human VEGF-A	R&D Systems	QVE00B; RRID: AB_2818940
Human PlGF	R&D Systems	SPG00
Human osteopontin	R&D Systems	DO ST00
Human Erythropoietin (EPO)	R&D Systems	DEP00
Human ceruloplasmin	Eagle Biosciences (Amherst, NH 03031)	HCP 31-K01
Rat VEGF-A	R&D Systems	RRV00; RRID: AB_2827538
Rat osteopontin	R&D Systems	MOST00
mirVana PARIS kit	Ambion	AM1556
miScript SYBR Green PCR This product is discontinued as of May 1^st^, 2021 and should be replaced by the miRCURY LNA SYBR® Green PCR Kits according to Qiagen.	Qiagen	339345

**Experimental models: Organisms/strains**		

Male Sprague-Dawley rats	Charles River	CD® (Sprague Dawley) Rats; RRID: MGI:5651135

**Oligonucleotides**		

Hsa-miR-30a	Applied Biosystems	4427975-000417

**Software and algorithms**		

R statistical software version 4.0.3	https://www.r-project.org/	RRID: SCR_001905

### Resource availability

#### Lead contact

Further information and requests for resources and reagents should be directed to and will be fulfilled by the lead contact, Nicolas Vodovar (nicolas.vodovar@inserm.fr).

#### Materials availability

This study did not generate new unique reagents.

#### Data and code availability

• Data used and/or analyzed during the current study are available from the lead contact upon reasonable request.
• This paper does not report original code
• Any additional information required to reanalyze the data reported in this paper is available from the lead contact upon request.

### Experimental model and study participant details

#### Study population

This study was performed according to the current revision of the Helsinki Declaration, approved by the Institutional Review Board (IRB 00006477; agreement: 16-035), and registered at clinicaltrials.gov under the NCT01374880 identifier. The study population was previously described^2^^,^^29^ and its main characteristics are reported in Table S1. Briefly, it consisted in 73 patients with HFrEF who were transitioned from an Angiotensin-converting enzyme inhibitor/Angiotensin receptor blocker to sacubitril/valsartan as per ESC guidelines.^33^ Blood samples were collected before the first administration of S/V (D0), at one (D30) and three months (D90) after the initiation of S/V. For some patients, blood samples were also collected one (D7) and two (D14) weeks after the initiation of S/V. Of note, the number of measurements may vary due to plasma exhaustion.

#### Plasma sampling

Venous blood samples were collected in tubes containing EDTA. Blood samples were immediately centrifuged at 3,500 rpm for 15 min at 4°C, frozen and stored at −80°C until further use.

#### Data collection

Hemoglobin, hematocrit, and red blood cell count (RBC) were performed on routine blood tests and collected from patient files. The estimated glomerular filtration rate (eGFR) was calculated using the 2021 update of the CKD-EPI equation.^34^

#### Animal experiments

Animal care and experimental procedures complied with the European Communities Council Directive (CEE 86/609/EEC), EU Directive 2017/32/EU, and French Departmental Direction of Animal Protection (2019-11 #1027). Male Sprague-Dawley rats (n = 6), aged 3 months, were from Charles River and housed in light- (on from 0800 to 2000 h) and temperature (21°C–24°C)-controlled testing rooms, with food and water available *ad libitum*. After acclimation, the same rats were placed in an isobaric hypoxic environment by gradually replacing oxygen with nitrogen at a rate of 0.5% per 6 h to reach 15% oxygen. After a week in hypoxic conditions, oxygen was gradually restored back to 20% at a rate of 0.5% per 6 h, and rats were acclimated back to normal atmosphere for a week. The level and time of hypoxia were chosen to be less severe than the chronic hypoxia model used to induce pulmonary hypertension (O2 level at 10% for at least 2 weeks). Males were chosen to reflect the gender bias of the cohort (Table S1). Sample size estimation was calculated on trial data obtained from 6 animals subjected to the protocol, using changes in K709ac-HIF1α as primary endpoint given that K709ac-HIF1α is the most direct hypoxia-induced factor. We therefore calculated sample size for within factor comparison (repeated measure ANOVA) with one group and 3 time points, using α = 0.05, power (1 – β) = 0.90, and a size effect calculated at 0.89 by the following formula:

$$f=\frac{\sqrt{\frac{\sum_{n=1}^{n=k}{\left(\mu_{n}-\mu \right)}^{2}}{k}}}{\sigma }$$

where μ_k_ is the mean at each timepoint, μ is the overall mean, k the number of time points, and σ the overall standard deviation. The calculation confirmed a sample size of 6 animals and that there was no need for increasing the number of animals in full compliance with the 3R.

#### Plasma sampling from rats

Venous blood was sampled after initial acclimation, after acclimation in hypoxic conditions, and after acclimation following the return to normoxia. Samples were collected from the tail vein under local anesthesia with lidocaine into ice-cold heparin-containing tubes maintained on ice. Blood samples were immediately centrifuged at 2,000 g for 10 min at 4°C, and plasma was aliquoted, immediately frozen, and stored at −80°C until use. Animals were euthanized using pentobarbital and lidocaine.^35^

### Method details

#### Biomarker quantifications

##### Plasma proteins quantification

All reagents were obtained from Sigma-Aldrich unless otherwise stated. Human VEGF-A, PlGF, osteopontin, and EPO were measured in plasma samples with standard enzyme-linked immunosorbent assay (ELISA) kits (refs QVE00B, SPG00, DO ST00, and DEP00, R&D Systems, Minneapolis, MN, respectively). Serum ceruloplasmin was measured by an ELISA (Eagle Biosciences, ref HCP31-K01) adapted on a Beckman Array Protein System (Beckman Instruments Inc, Brea, CA, USA). Rat VEGF-A and osteopontin were quantified in plasma samples using standard ELISAs (refs RRV00 and MOST00, R&D Systems, Minneapolis, MN, respectively). Rat EPO and ceruloplasmin were measured by radioimmunoassay using commercially available antibodies (refs sc-5290 and sc-365205, Santa Cruz Biotechnology).

##### Plasma enzymatic activity

Ceruloplasmin oxidase activity was measured, as previously described^36^: briefly, plasma samples containing 2 mg protein were added in duplicate to plastic cuvettes containing 0.6 mL of 0.1M acetate buffer (pH 6.0), 0.3 mL 0.25% *p*-phenylenediamine (PPD) in 0.1M acetate buffer, which had been equilibrated at 37°C for 5 min; blank samples containing 2 mg plasma protein, 0.3 mL 0.1M acetate buffer, 0.3 mL 0.1% NaN_3_ in acetate buffer, and 0.3 mL 0.25% PPD in acetate buffer were prepared for each test sample; the reaction mixtures were incubated at 37°C, and the absorbance read at 530 nm after 10 and 40 min; ceruloplasmin oxidase activity was calculated in terms of oxidase units, where one oxidase unit = (Abs40min - Abs10min) × 1000.

##### Quantification of ProBNP glycosylation by mass spectrometry

ProBNP glycosylation at threonine 71^32^ and histone lactylation^14^ were measured by mass spectrometry (LC-MS/MS) as previously described. HIF-1α K709ac was measured by MS/MS. Proteins were reduced with 25 mM Tris(2-carboxyethyl)phosphine hydrochloride (TCEP) in denaturing urea buffer, alkylated with iodoacetamide, and diluted with digest buffer (100 mM Tris pH 8.0 + 1 mM CaCl_2_) to reach 4 M urea, before being digested with 1:50 Lys-C (Custom Biotech, ref. 11058533103). The digestion was diluted with digest buffer to reach 0.8 M urea and digested with trypsin (Promega; 1:50). The digests were then desalted using SepPak-C18 SPE cartridges, dried and resuspended in 80% Acetonitrile, 0.1% TFA. Peptides were separated using a Dionex Ultimate 3000 HPLC system equipped with a HILIC (hydrophilic interaction liquid chromatography) column, using a similar protocol to the hSAX method described previously.^37^ The peptide fractions were desalted using SepPak-C18 SPE plates and then resuspended in 5% formic acid for LC-MS/MS analysis. For Trypsin+Lys-C double digests, peptide chromatography was performed using a Dionex RSLCnano HPLC. Peptides were injected directly onto a 75 μm × 50 cm PepMap-C18 column using the following mobile phases: 2% acetonitrile +0.1% formic acid (Solvent A) and 80% acetonitrile +0.1% formic acid (Solvent B). The linear gradient began with 5% A to 35% B over 220 min with a constant flow rate of 200 nL/min. The peptide eluent flowed into a nanoelectrospray emitter at the front end of a Q-Exactive (quadrupole Orbitrap) mass spectrometer (Thermo Fisher). A typical “Top10” acquisition method was used and an in-house developed software inspired by^38^ was used to evaluate peptide identifications and abundance ratios.

##### miRNA quantification by qPCR

miR-30a was measured by qPCR. Total RNA was extracted from plasma samples using the mirVana PARIS kit (Ambion, Thermo Fisher Scientific, St-Quentin en Yvelines, France). Hemolyzed samples were avoided since hemolysis was reported to alter miR plasma content.^39^ Spiked-in *synthetic C. elegans* miRNA controls (cel-miR39, 54 and 238; Qiagen, Courtaboeuf, France) were added to samples for correction of extraction efficiency. After DNAse treatment, RNAs were reverse transcribed with the miScript reverse transcription kit (Qiagen). cDNA was diluted 10-fold before quantitative PCR with the miScript SYBR Green PCR kit (Qiagen). A control without reverse transcriptase and a control without RNA was added to each PCR plate to ensure the absence of contaminating DNA and to check for non-specific amplification, respectively. Primers and probes for miR-30a were purchased from Applied Biosystems. Expression values were normalized using the mean Ct of the spiked-in controls and calculated with the 2^−ΔCt^ formula.

##### Quantification of metabolites by HPLC and mass spectrometry

Plasma tryptophan was measured by HPLC with coulometric detection as previously described.^40^ *p*-cresyl sulfate, and indoxyl sulfate were measured using LC-MS/MS with stable isotopic dilution as previously described.^41^ The determination of unconjugated *p*-cresol by LC-MS/MS sample preparation included derivatization with dansyl chloride according to.^42^

### Quantification and statistical analysis

Statistical analyses were performed using R version 4.0.3.^43^ Normality was assessed using the Shapiro-Wilk test and continuous variables are expressed as median and interquartile range. Longitudinal data were analyzed using repeated measures ANOVA on log-transformed values using a linear mixed model generated with the *lme* function of the *nlme* package in R.^44^ Post-hoc analysis was performed using the paired Student’s *t* test corrected for multiple comparisons (holm). Comparisons between baseline and D7 or D14 were performed using the Wilcoxon signed-rank tests. Relationships between continuous variables were assessed using Spearman rank correlation (***ρ***). A p-value *<* 0.05 was considered statistically significant.

### Additional resources

The human study was registered at clinicaltrials.gov under the NCT01374880 identifier.
