# Supplementary material for: Impact of sacubitril/valsartan on cardiac and systemic hypoxia in chronic heart failure
Source: iScience. 2023 Nov 23;27(1):108520. doi: 10.1016/j.isci.2023.108520 (PMC10755360; doi:10.1016/j.isci.2023.108520)
Supplement: Document S1. Figures S1–S6 and Table S1 [file mmc1.pdf]

iScience, Volume *27*

Supplemental information

Impact of sacubitril/valsartan on cardiac and systemic hypoxia in chronic heart failure

Hélène Nougué, François Picard, Alain Cohen-Solal, Damien Logeart, Jean-Marie Launay, and Nicolas Vodovar

Table S1: Baseline characteristics of the study population (n=73) originally published in [2], Related to STAR Methods.

**Patient characteristics**	
Age (year)	65 [56; 72]
Men	61 (84%)
Body mass index (kg/m^2)	25.1 [23.0; 28.0]
Diabetes	16 (22%)
Atrial fibrillation	25 (34%)
Ischemic heart disease	40 (55%)
Last decompensation (months)	6 [3; 25]
Systolic blood pressure (mmHg)	114 [102; 125]
Diastolic blood pressure (mmHg)	67 [60; 77]
eGFR (ml/min/1.73m^2)	62 [48; 84]
NYHA classification	
I	2 (3%)
II	36 (49%)
III	33 (45%)
IV	2 (3%)
**Medication**	
Furosemide	62 (85%)
ACEi or ARB	72 (98%)
Beta-blocker	68 (73%)
MRA	58 (73%)
**Plasma biomarkers**	
Creatinine (µmol/l)	103 [87; 124]

BNP (ng/L	370 [193; 702]
NT-proBNP (ng/L	1201 [571; 1997]
hsTnI (ng/L)	14.3 [9.4; 20.7]
sNEP activity (pmol/ml/min)	340 [254; 445]
sNEP concentration (pg/mL)	241 [205; 303]
ANP_{1-28} (pM)	37.4 [23.5; 55.0]
GLP-1 (pmol/L)	3.0 [2.6; 3.1]
Substance P (ng/L)	36 [27; 44]
Fructosamine (µM)	265 [255; 296]
sST2 (ng/mL)	27.3 [21.3; 38.4]
sCD146 (ng/mL)	403 [317; 560]

Legend: eGFR estimated glomerular filtration rate, NYHA New York Heart Association, ACEi: angiotensin-converting enzyme inhibitor, ARB: angiotensin receptor blocker, MRA mineralocorticoid receptor antagonist, BNP: B-type natriuretic peptide, NT-probNP: N-terminal fragment of proBNP, hsTnI: high-sensitive troponin I, sNEP: soluble néprilysine, ANP: A-type natriuretic peptide, GLP-1: glutathione-like peptide 1, sST2: soluble form of the IL-33 receptor, sCD146: soluble cluster of differentiation 146. Variables are expressed as number (percentage) or median [interquartile range], as appropriate.

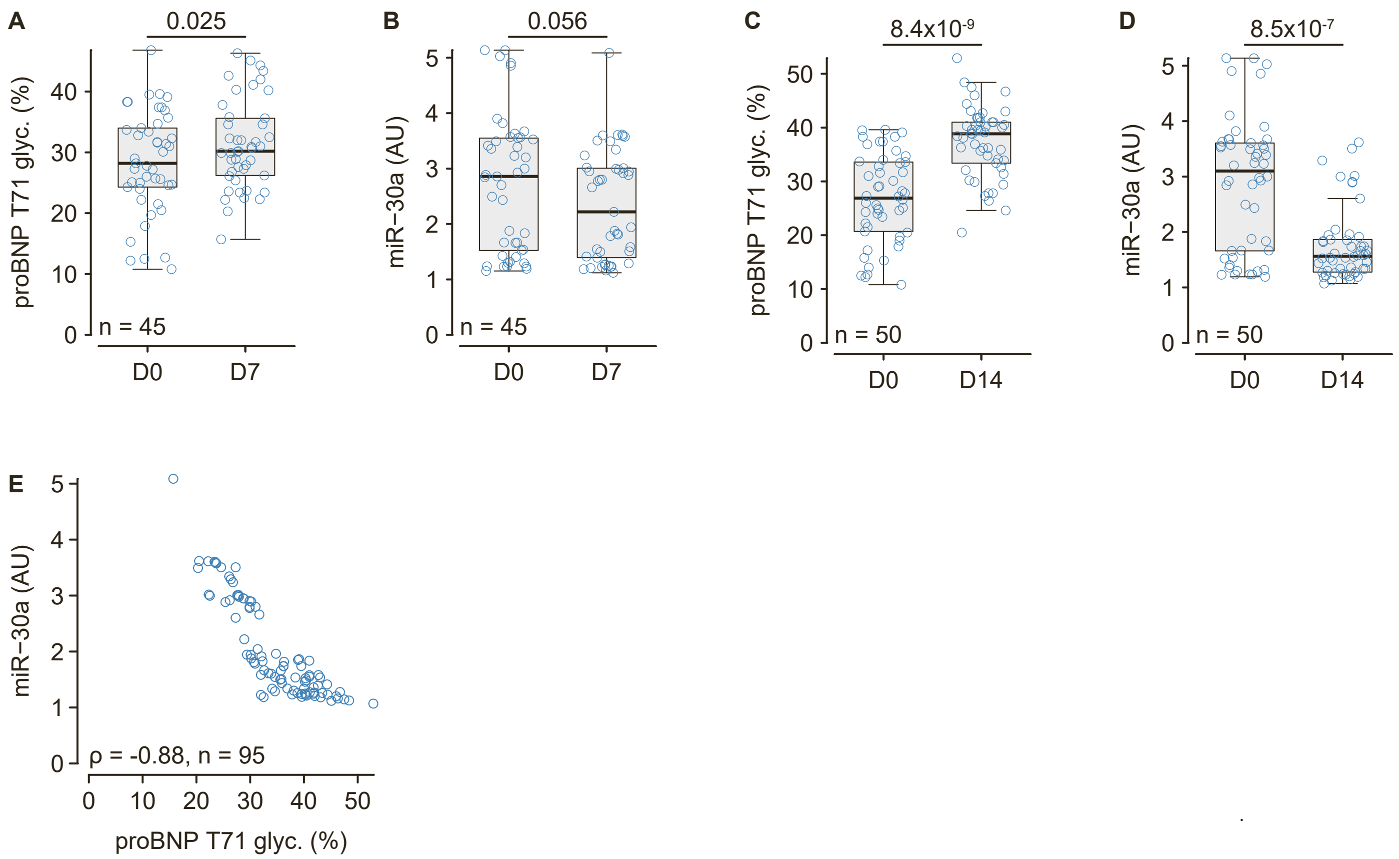

Figure S1: proBNP glycosylation at T71 and miR-30a *in vivo*, Related to Figure 1. Comparison of plasma levels of proBNP T71 glycosylation miR-30a (**A** and **C**) and miR-30a (**B** and **D**) between baseline (D0) and D7 (**A-B**), and between baseline and D14 after the introduction of S/V (**C-D**). **E)** Relationship between miR-30a and proBNP T71 glycosylation for samples collected at D7 and D14. Variables in **A-D** were analyzed using the Wilcoxon signed-rank test.Correlations between variables were estimated with Spearman rank correlation and expressed by the correlation coefficient (ρ). The number of subjects is indicated in each panel.

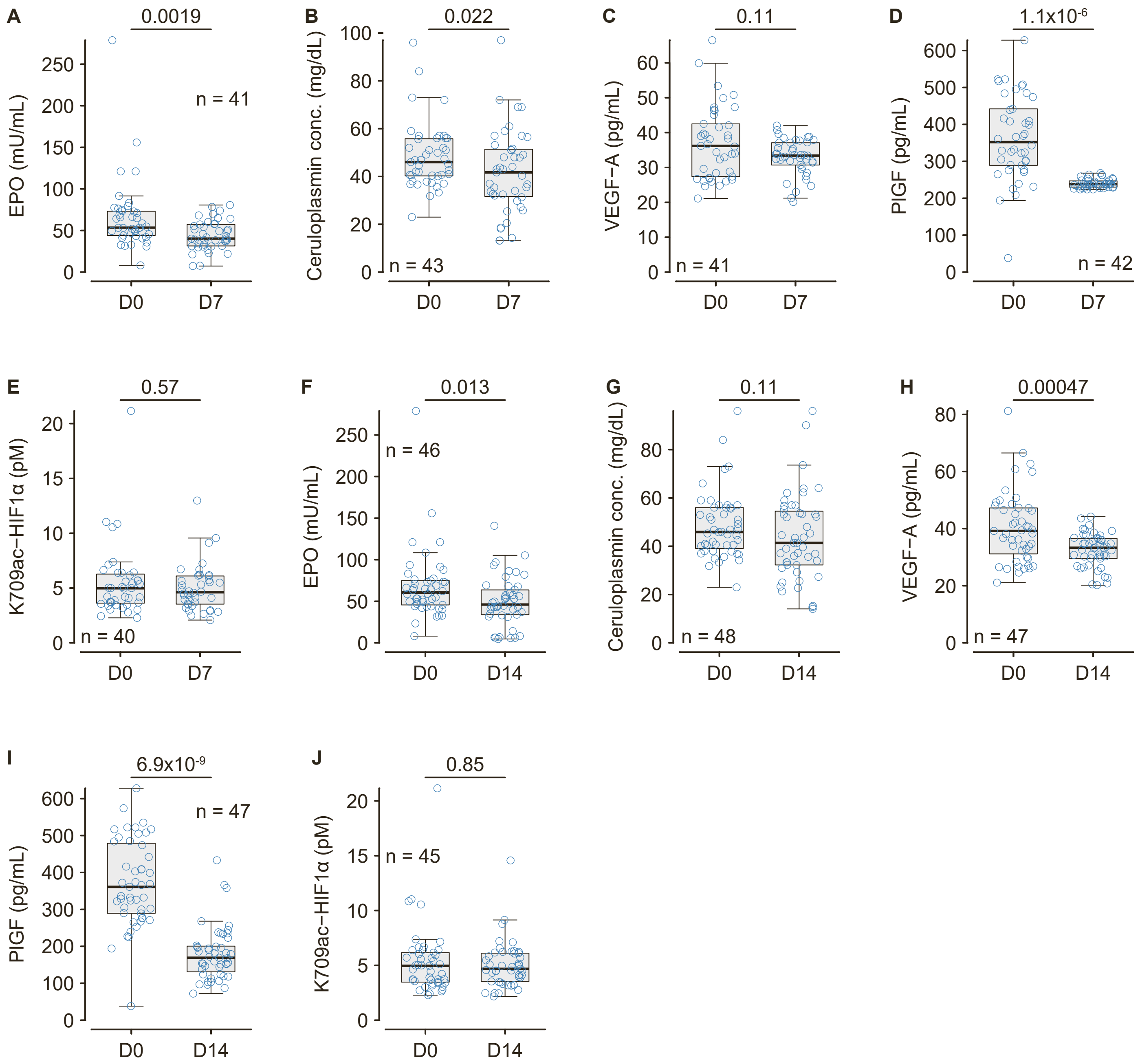

Figure S2: Early evolution of HIF1α-dependent biomarkers, Related to Figure 2. Comparison of plasma levels of EPO (**A** and **F**), ceruplasmin (**B** and **G**), VEGF-A (**C** and **H**), PlGF (**D** and **I**), and HIF1α-K709ac (**E** and **J**) between baseline (D0) and D7 (**A-D**), and between baseline and D14 after the introduction of S/V (**E-J**). Variables were analyzed using the Wilcoxon signed-rank test. The number of subjects is indicated in each panel.

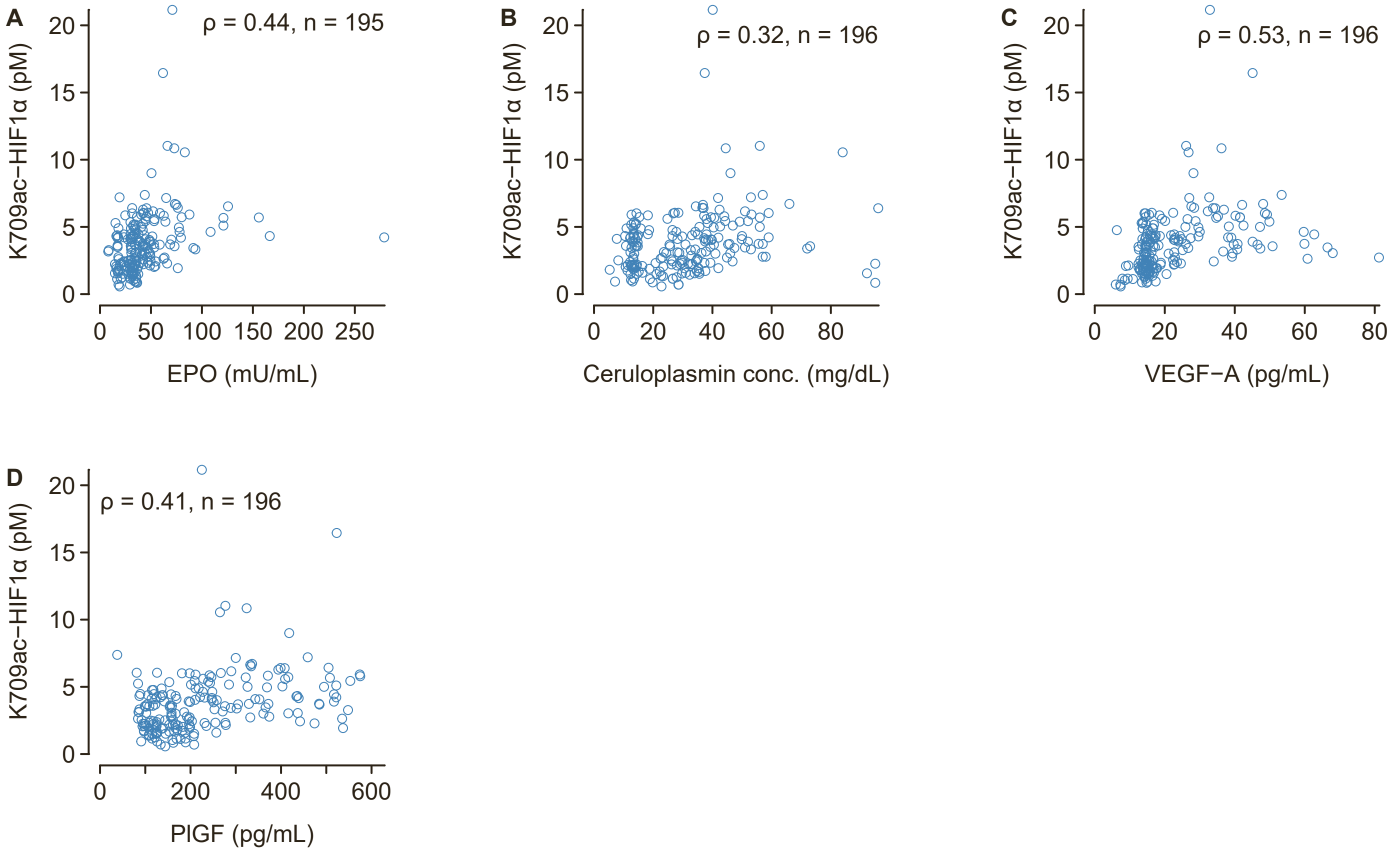

Figure S3: Relationships between HIF1α-K709ac and HIF1α-dependent biomarkers, Related to Figure 2. Relationships between HIF1α-K709ac and EPO (**A**), ceruloplasmin concentration (**B**), VEGF-A (**C**), and PlGF (**D**). Correlations between variables were estimated with Spearman rank correlation and expressed by the correlation coefficient (ρ). The number of subjects is indicated in each panel.

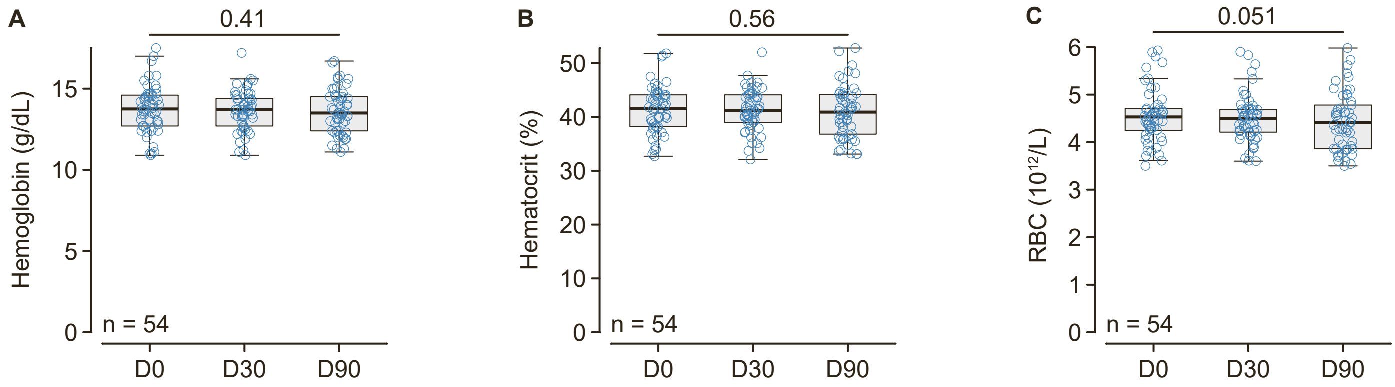

Figure S4: Evolution of oxygen transport capablility, Related to Figure 2. Hemoglobin levels (**A**), hematocrit (**B**), red blood cell count (RBC, **C**) at baseline, D30 and D90 after the introduction of S/V. Variables were analysed using repeated-measure ANOVA on log-transformed data followed by the paired Student's t-test corrected for multiple comparisons (holm). The number of subjects is indicated in each panel.

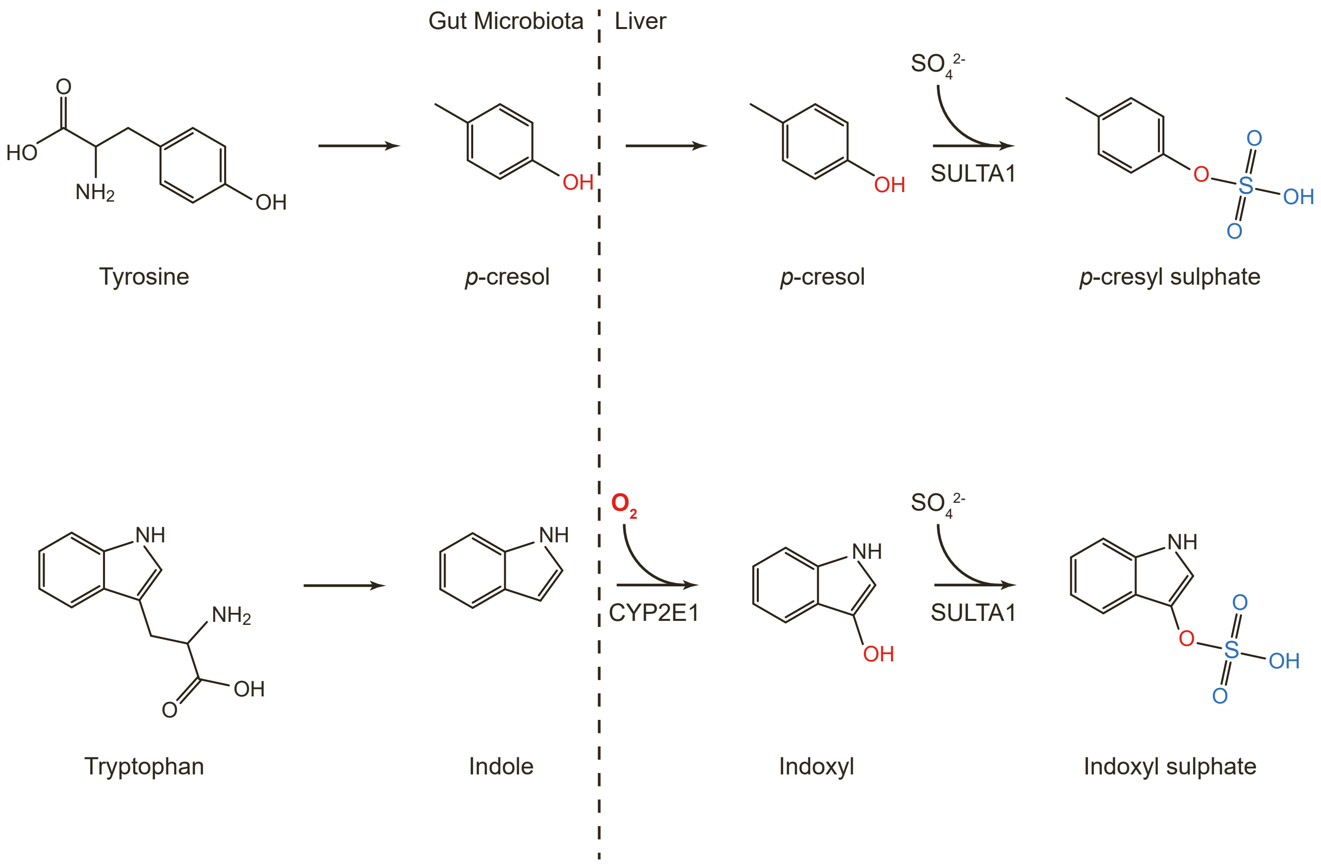

Figure S5: Microbial metabolism of tyrosine and tryptophan, Related to Figure 4. Schematic representation of the production of *p*-Cresyl Sulphate from Tyrosine and Indoxyl Sulphate from Tryptophan. The major metabolic difference between the production of *p*-Cresyl sulphate and indoxyl sulphate is the hydroxylation step between indole and indoxyl by cytochrome CYP2E1, which is dependent on molecular oxygen.

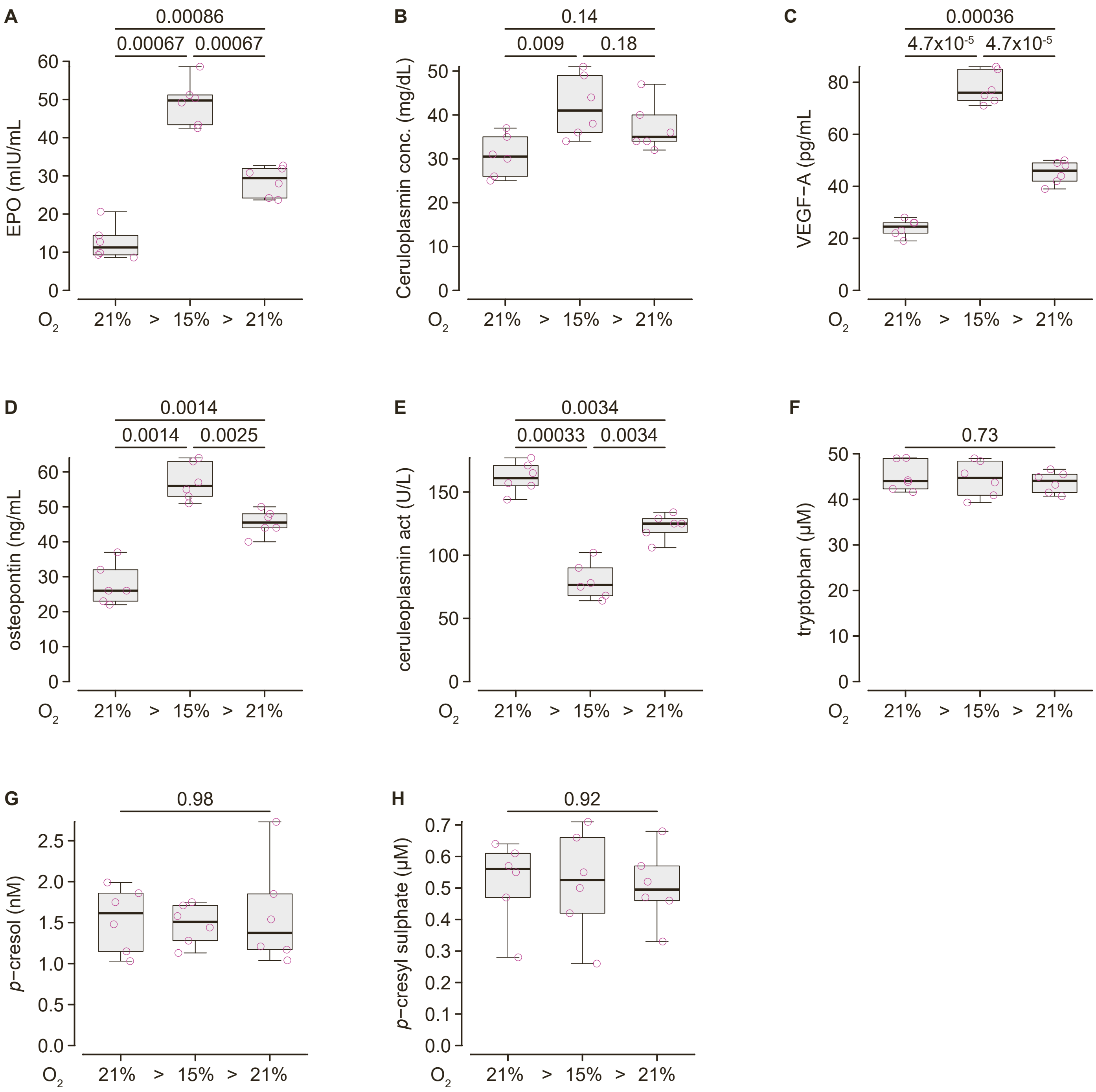

Figure S6: Evolution of hypoxia biomarkers in rats subjected to isobaric hypoxia, Related to Figure 5. Plasma levels of erythropoietin (EPO, **A**), ceruloplasmin (**B**), vascular endothelial growth factor A (VEGF-A, **C**), osteopontin (**D**), ceruloplasmin activity (**E**), tryptophan (**F**), *p*-cresol (**G**), and *p*-cresyl sulphate (**G**) in rats under normoxic conditions (20% O2), after one week in isobaric hypoxia (15% O2), and one week after returning to normoxia (20% O2). Variables were analysed using repeated-measure ANOVA on log-transformed data followed by the paired Student's t-test corrected for multiple comparisons (holm). There were 6 rats in the experiment that were followed longitudinally throughout the study.
